# Association between -174G>C polymorphism in the IL-6 promoter region and the risk of obesity: A meta-analysis: Erratum

**DOI:** 10.1097/MD.0000000000012616

**Published:** 2018-09-21

In the article, “Association between -174G>C polymorphism in the IL-6 promoter region and the risk of obesity: A meta-analysis”,^[1]^ which appeared in Volume 97, Issue 17 of *Medicine*, the affiliation of Dr. Man Hu is unclear and should appear as:

Department of Intergarted Traditional Chinese and Western Medicine, Union Hospital, Tongji Medical College, Huazhong University of Science and Technology, Wuhan,430022, China.
